# Supplementary material for: Cryo-EM structure of an early precursor of large ribosomal subunit reveals a half-assembled intermediate
Source: Protein Cell. 2018 Mar 19;10(2):120–30. doi: 10.1007/s13238-018-0526-7 (PMC6340896; doi:10.1007/s13238-018-0526-7)
Supplement: Supplementary file 3 — Supplementary material 3 (PDF 4254 kb) [file 13238_2018_526_MOESM3_ESM.pdf]

Cryo-EM structure of an early precursor of large ribosomal subunit reveals a half-assembled intermediate

Dejian Zhou, Xing Zhu, Sanduo Zheng, Dan Tan, Meng-Qiu Dong and Keqiong Ye

Supplementary Materials:
Figure S1-S6
Table S1-S3
Dataset 1-2

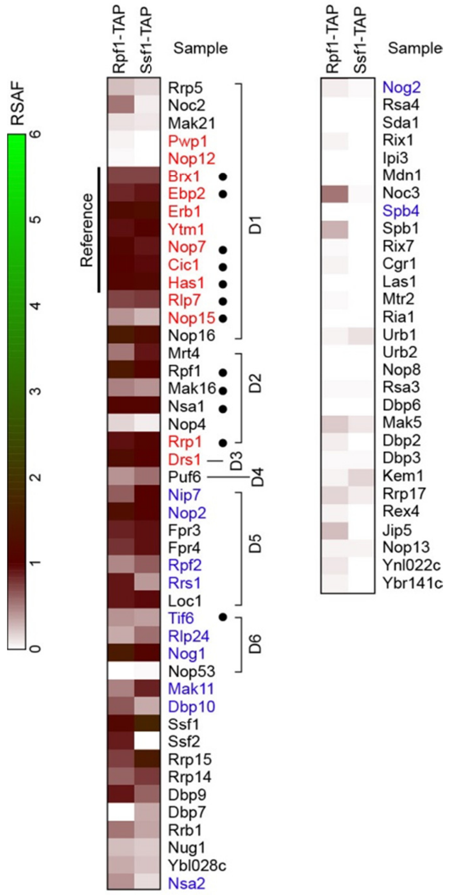

Figure S1. Heatmap of pre-60S AFs in Rpf1-TAP particle.

The proteins identified by mass spectrometry are color-coded according to their RSAF values normalized against the reference proteins Brx1, Ebp2, Erb1, Ytm1, Nop7, Cic1 and Has1. The previously reported Ssf1-TAP data are included for comparison (Chen et al., 2017). The AFs on the top of the list are arranged by their association order to pre-27S rRNA fragments ending at domains I to VI (D1-D6). A3-factors and B-factors are colored red and blue, respectively. Solid circles mark 12 AFs modeled in the Rpf1-TAP pre-60S structure.

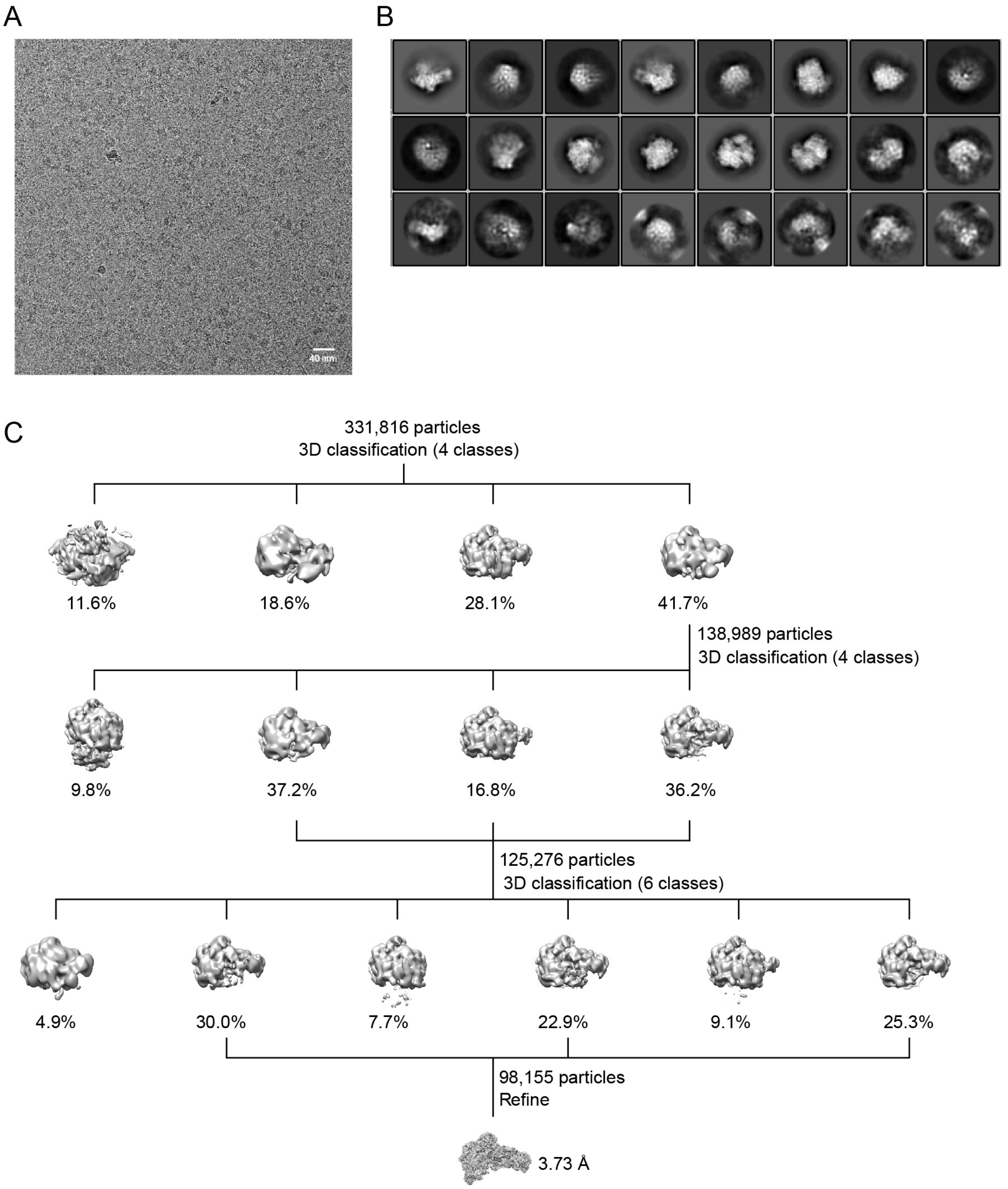

Figure S2. Cryo-EM analysis of Rpf1-TAP particles.

(A) Electron micrograph of Rpf1-TAP particles. Bar = 40 nm. (B) 2D class averages from reference-free alignment. Some averages are from 80S and 60S ribosomes. Mask diameter = 400 Å (C) Flowchart of 3D classification and refinement.

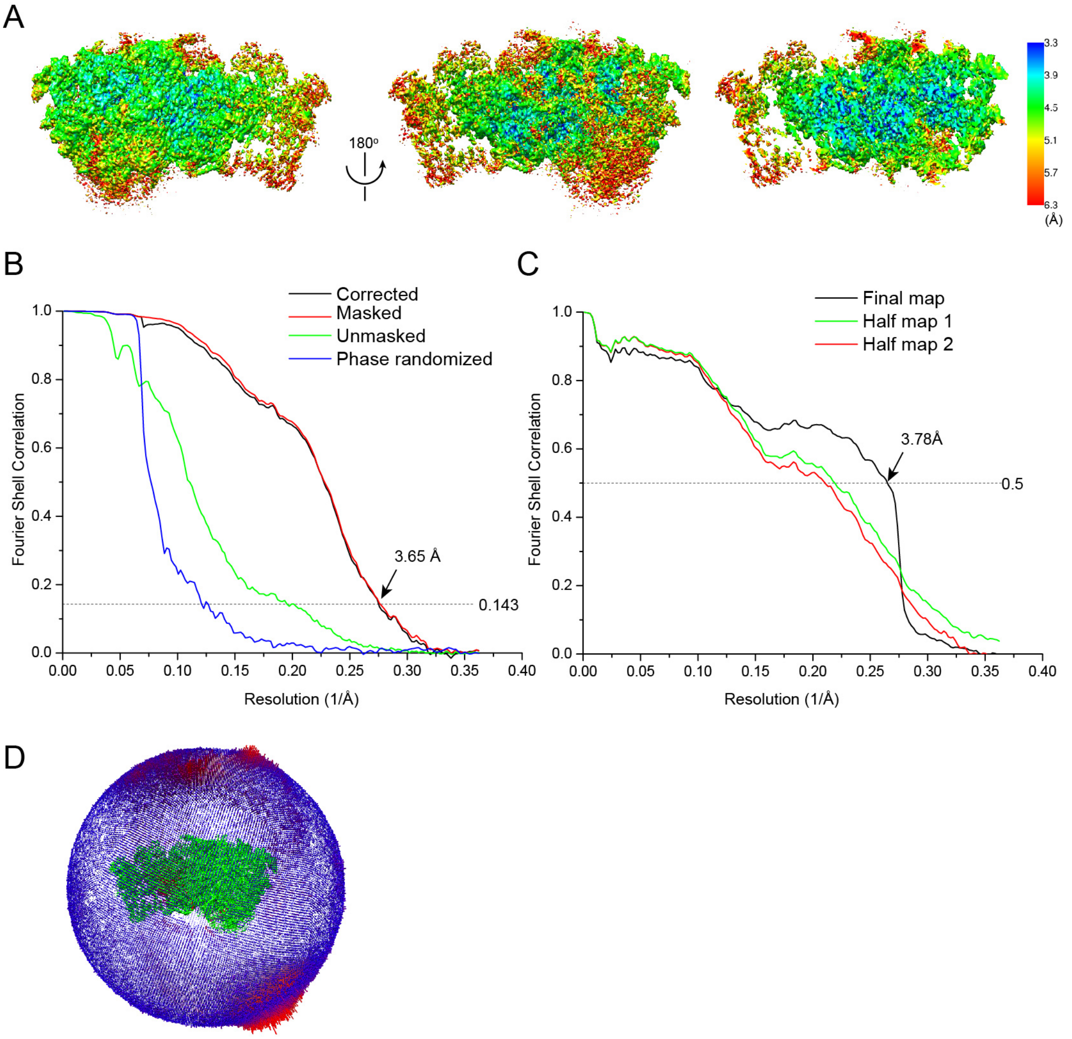

Figure S3. Quality of the cryo-EM map of Rpf1-TAP pre-60S.

(A) Local resolution cryo-EM map. The left and middle panels are surface views in opposite directions and the right panel is a cut-through view showing the interior of the structure. (B) FSC curves calculated in RELION for the corrected (black), masked (red), unmasked (green) and phase-randomized (blue) maps. The resolution is 3.65 Å according to the FSC=0.143 criterion. (C) FSC curves of model versus map and cross-validation of the Rpf1-TAP pre-60S model. Black: FSC between the refined structure and the final postprocessed map. Green: FSC between the re-refined model and half-map 1 it was refined against. Red: FSC between the re-refined model and the other half-map it was not refined against. The resolution is 3.78 Å according to the FSC=0.5 criterion. (D) Angular distribution of particles used for the final reconstruction. Each bar represents one orientation. The height of the bar is proportional to the number of particles in that orientation.

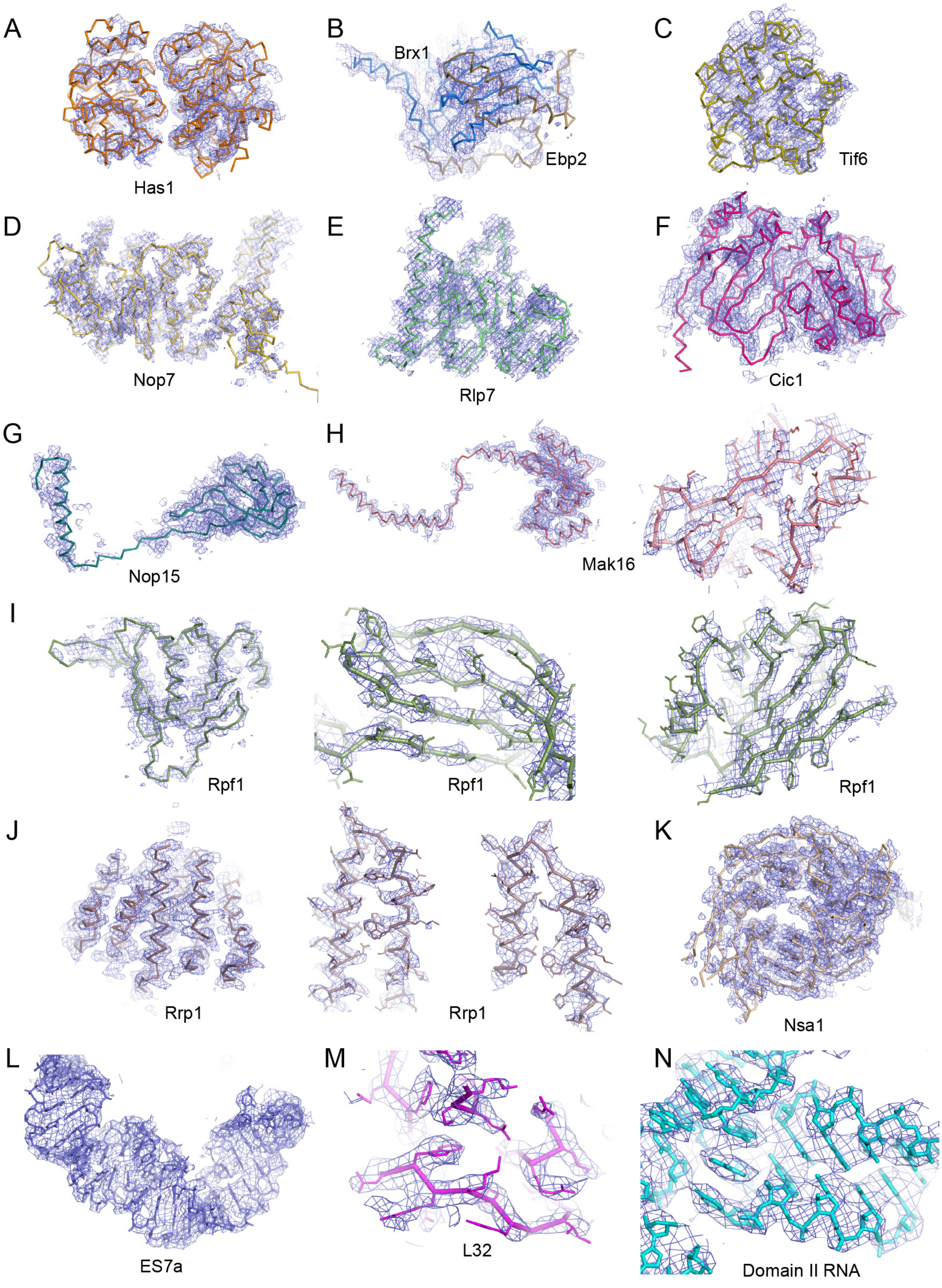

Figure S4. Representative cryo-EM densities with fitted structural models.

(A) Has1. (B) The Brx1-Ebp2 complex. (C) Tif6. (D) Nop7. (E) Rlp7. (F) Cic1. (G) Nop15. (H) Mak16. (I) Rpf1. (J) Rrp1. (K) Nsa1. (L) ES7a. (M) L32. (N) Domain II RNA. To reduce noise, the Gaussian filtered map (standard deviation =1) is displayed for Has1, Brx1/Ebp2 and Tif6. Proteins are shown as Cα traces and RNAs as sticks. Parts of the Rpf1, Mak16, Rrp1 and L32 structures are also displayed with side chains to illustrate the quality of density at the core region.

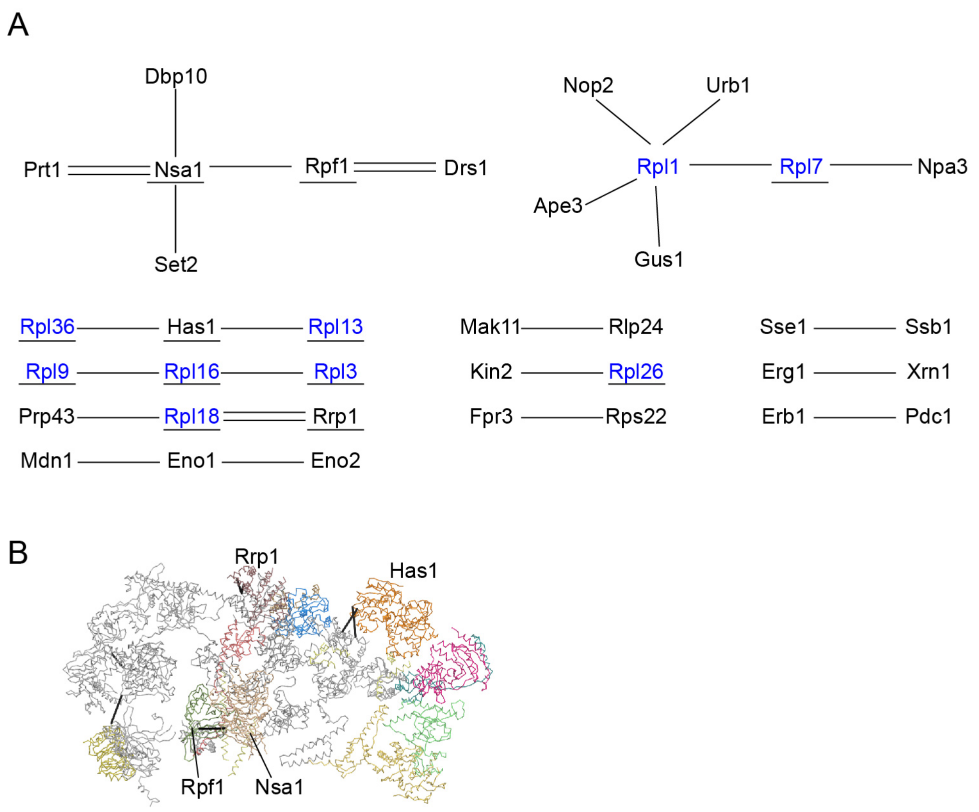

Figure S5. CXMS of Rpf1-TAP particle

(A) Schematic of intermolecular crosslinks. Each crosslink is represented by a line. Ribosomal proteins are colored blue. The proteins modelled in the Rpf1-TAP pre-60S structure are underlined. (B) Mapping of intermolecular crosslinks to the Rpf1-TAP pre-60S structure. The Cα atoms of each crosslinked lysine pair are connected by a line. When a crosslinked lysine is not modeled in the structure, its closest modeled residue in the primary sequence is used for drawing. Ribosomal proteins are colored in grey and AFs are color coded. Crosslinked AFs are labeled.

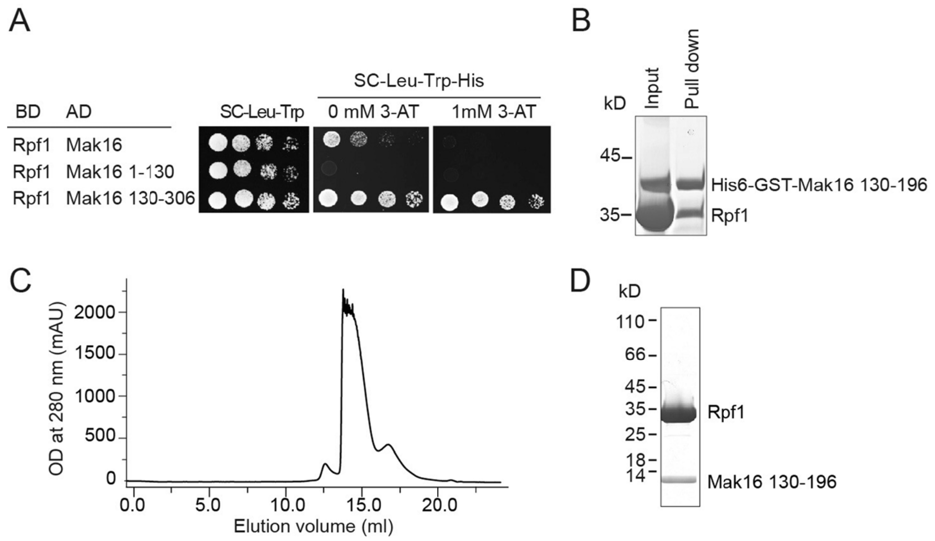

Figure S6. Rpf1 binds a short sequence of Mak16.

(A) Yeast two-hybrid assay. Rpf1 was fused to the GAL4 DNA-binding domain (BD) as bait. Mak16 and its fragments were fused to the GAL4 activation domain (AD) as prey. Yeast AH109 cells were co-transformed with bait and prey plasmids, five-fold serially diluted and spotted in plates with Synthetic Complete (SC) medium lacking Leu and Trp as growth controls and in plates with SC medium lacking Leu, Trp and His and containing the indicated concentration of 3'-amino-1,2,4-triazole (3-AT) to examine the prey-bait interaction. (B) GST pull-down assay. Rpf1 was incubated with a His_6-GST-tagged Mak16 fragment containing residues 130-196 and pulled down with glutathione Sepharose beads. The input and eluate were examined by SDS-PAGE and Coomassie blue staining. The positions of molecular standards are indicated on the left. (C) Gel filtration profile of the co-expressed and co-purified complex of Rpf1 and Mak16 130-196 in a Superdex 200 column. (D) SDS-PAGE gel of the peak fraction in C.

Table S1. Modeling of the Rpf1-TAP pre-60S structure

Components	Alias	Length	Chain	Modeling	Template	Remarks & crosslinks for protein assignment
25S rRNA		3396	A	Domains I, II, VI (1501 nt)	3JCT	Manual building of ES7a
5.8S rRNA		158	B	Residues 1-156	3JCT	
ITS2 RNA		232	C	Residues 1-59, 227-232	3JCT	
Rpl3	uL3	387	F	Residues 12-227, 268-387	3JCT	
Rpl4A	uL4	362	G	Residues 2-362	3JCT	
Rpl6A	eL6	176	I	Residues 8-109, 129-176	3JCT	
Rpl7A	uL30	244	J	Residues 6-244	3JCT	Manual building of residues 6-21
Rpl8A	eL8	256	K	Residues 69-119, 125-235	3JCT	
Rpl9A	uL6	191	L	Residues 1-191	3JCT	
Rpl13A	eL13	199	P	Residues 21-128	3JCT	
Rpl14A	eL14	138	Q	Residues 2-138	3JCT	
Rpl15A	eL15	204	R	Residues 2-68, 97-204	3JCT	
Rpl16A	uL13	199	S	Residues 3-199	3JCT	
Rpl17A	uL22	184	T	Residues 10-124, 140-160	3JCT	
Rpl18A	eL18	186	U	Residues 15-148	3JCT	
Rpl20A	eL20	172	W	Residues 2-171	3JCT	
Rpl26A	uL24	127	c	Residues 2-126	3JCT	
Rpl32	eL32	130	i	Residues 5-128	3JCT	
Rpl33A	eL33	107	j	Residues 2-107	3JCT	
Rpl35A	uL29	120	l	Residues 3-119	3JCT	
Rpl36A	eL36	100	m	Residues 32-98	3JCT	
Rpl37A	eL37	88	n	Residues 14-85	3JCT	
Rlp7		322	D	Residues 55-105, 127-322	3JCT	
Nop15		220	E	Residues 88-220	3JCT	
Cic1	Nsa3	376	H	Residues 31-51, 71-305	3JCT	
Nop7		605	M	Residues 1-267, 351-396, 403-460	3JCT	
Tif6		245	N	Residues 1-226	3JCT	
Rpf1		295	O	Residues 88-177, 194-295	5WXL	Homology model and manual building<br>NSA1_14-RPF1_72
Nsa1		463	V	Residues 1-78,101-148, 151-416	5SUI	NSA1_14-RPF1_72
Rrp1		278	X	Residues 3-31, 38-74, 88-140, 145-158, 165-186, 198-243.		Manual building<br>RPL18A_133-RRP1_239<br>RPL18A_133-RRP1_232
Has1		505	Y	Residues 41-253, 260-484	4TYW	Homology model<br>HAS1_110-RPL13A_117<br>HAS1_188-RPL36A_16

Mak16		306	Z	Residues 2-170		Manual building
Brx1		291	a	Residues 26-65, 70-195, 210-255	5Z1G	
Ebp2		427	b	Residues 195-276	5Z1G	
Unassigned			d	Poly-alanine chain (154 residues)		Manual building

Table S2. Statistics of data collection, structural refinement and model validation

	Rpf1-TAP pre-60S
**Data collection**	
EM equipment	FEI Titan Krios
Voltage (kV)	300
Detector	Falcon III
Grid	GiG R422
Micrographs	1983
Particles for 3D classification	331816
Pixel size (Å)	1.38
Defocus range (μm)	1-4
Electron dose (e^-/Å^2)	30
**Map refinement**	
Particles for refinement	98155
Overall resolution of map (Å)	3.65
Map sharpening B-factor (Å^2)	-71.4
**Model composition**	
Protein chains	32
Protein residues	6181
RNA chains	3
RNA bases	1722
**Structural refinement**	
Map CC (whole unit cell)	0.502
Map CC (around atoms)	0.750
RMSD Bonds (Å)	0.013
RMSD Angles (°)	1.240
**Validation (protein)**	
All-atom clashscore	35.48
Rotamer outliers (%)	0.29
Ramachandran plot favored (%)	87.71
Ramachandran plot allowed (%)	11.68
Ramachandran plot outliers (%)	0.61

Table S3. Data collection and refinement statistics of the Brx1-Ebp2 crystal structure

Crystal form	Native	Se-labeled
Data collection		
Space group	$P2_1$	$C2_1$
Cell dimensions		
a, b, c (Å)	80.9, 47.4, 100.7	100.3, 47.0, 71.9
α, β, γ (°)	90.0, 94.0, 90.0	90.0, 118.8, 90.0
Wavelength (Å)	0.97930	0.97930
Resolution range (Å)	30.0-2.3(2.34-2.30)	25.0-2.80(2.85-2.80)
Unique reflections	33644(1715)	7463 (348)
Redundancy	3.5(3.6)	5.9(6.2)
$\langle I \rangle / \langle \sigma(I) \rangle$	17.8(4.0)	20.3(4.5)
Completeness (%)	97.5(99.9)	99.7(99.4)
R_{merge}	0.099(0.382)	0.179(0.784)
Structure refinement		
Resolution range (Å)	25-2.3(2.36-2.3)	
No. reflections	33214	
No. atoms	5459	
Protein	5146	
Water	308	
Sulfate ion	5	
R_{work}	0.230(0.278)	
R_{free}	0.297(0.394)	
Mean B factor ($Å^2$)	25.96	
Rmsd bond length (Å)	1.091	
Rmsd bond angles (°)	0.008	
Ramachandran Plot		
Favored (%)	96.53	
Allowed (%)	3.14	
Outliers (%)	0.33	

Values in parentheses are for the data in the highest resolution shell.

Supplementary Dataset 1. Mass spectrometry data.

The SCPHR and RSAF values of identified proteins are displayed in two separate sheets. The RSAF is normalized against Brx1, Ebp2, Erb1, Ytm1, Nop7, Cic1 and Has1. The top rows include the summed SCPHR or RSAF values for 90S proteins, pre-40S proteins, pre-60S proteins, small subunit ribosomal proteins (RPS), large subunit ribosomal proteins (RPL) and total identified proteins, the total spectral counts (SpC) of reference proteins and the molar percentages of pre-60S AFs and RPLs over all detected proteins. (Excel table)

Supplementary Dataset 2. CXMS data of the Rpf1-TAP sample.

The crosslinked proteins, crosslinked peptides, spectral counts and best E-values are listed. The distance between the Cα atoms of crosslinked residues that belong to different proteins and are both modeled in the structure are shown. If a crosslinked lysine is not modeled, its closest modeled residue in the primary sequence was used for distance measurement. (Excel table)
